# Characteristics of the Cardiosplenic Axis in Patients with Fatal Myocardial Infarction

**DOI:** 10.3390/life12050673

**Published:** 2022-05-01

**Authors:** Maria Kercheva, Vyacheslav Ryabov, Andrey Trusov, Ivan Stepanov, Julia Kzhyshkowska

**Affiliations:** 1 Cardiology Research Institute, Tomsk National Research Medical Center, Russian Academy of Sciences, 111a Kievskaya Street, 634012 Tomsk, Russia; rvvt@cardio-tomsk.ru (V.R.); truan94@mail.ru (A.T.); i_v_stepanov@mail.ru (I.S.); 2 Central Research Laboratory, Siberian State Medical University, 2 Moscovsky Trakt, 634055 Tomsk, Russia; 3 Laboratory of Translational and Cellular Biomedicine Department, National Research Tomsk State University, 36 Lenin Avenue, 634050 Tomsk, Russia; 4 Department for Innate Immunity and Tolerance, Institute of Transfusion Medicine and Immunology, University of Heidelberg, 1-3 Theodor-Kutzer Ufer, 68167 Mannheim, Germany; julia.kzhyshkoska@googlemail.com

**Keywords:** myocardial infarction, cardiac remodeling, splenic macrophages, spleen, CD 68, stabilin-1, inflammation

## Abstract

Myocardial ischemia triggers neurohumoral activation of the cardiosplenic axis. In rodents, adverse outcomes occur upon prolonged entrance of mononuclear cells from the spleen into myocardial tissue. The purpose of this study is to assess the features of spleen structure in patients with fatal myocardial infarction (MI), the dynamics of macrophage infiltration of the spleen and its relationship with cardiac macrophage infiltration and unfavorable outcomes. Using immunohistochemistry techniques, we analyzed the macrophage infiltration of the spleen and myocardium sections collected from patients (*n* = 30) with fatal MI. The spleen of the patients was decreased and showed a predominance of red pulp with a high concentration of CD68+ and stabilin-1+ cells. The white pulp contained many medium and small follicles and a lower concentration of CD68+ and stabilin-1+ cells, which was comparable to that in the infarct area of the myocardium. The concentration of CD68+ and stabilin-1+ cells increased in the myocardium in the late period of MI, but did not show any dynamics in the spleen. A high number of CD68+ cells in the red pulp and reduced concentration of stabilin-1+ cells in the white pulp were associated with unfavorable post-infarction outcomes. These fundamental findings could be a basis for the development of new personalized therapeutic and diagnostic approaches for the treatment of MI and its complications.

## 1. Introduction

Myocardial infarction (MI) is followed by the development of post-infarction heart failure (HF) and an unfavorable prognosis for one in every six patients [1]. Post-infarction HF is caused by adverse left ventricular remodeling (LVR), which includes both geometry impairment and worsening of the left ventricular (LV) pumping function, as well as changes of the cellular and molecular composition of the myocardium [2]. Many attempts have been made to describe the mechanism of formation and progression of post-infarction cardiac remodeling, but there has been no success in attempts to reverse or to prevent it [3,4].

The involvement of the innate immune system in the development of adverse LVR has been one of the promising areas in cardiology and has been intensively studied over the past decade [5]. Monocytes/macrophages are the key participants in this pathological process, and are of particular interest as they can be used for personalized targeted therapy for MI and its outcomes. A biphasic inflammatory response to myocardial ischemia is induced by the activation of embryonic-derived macrophages that reside within tissues, as well as by the involvement of monocytes from the physiological pool [6]. The spleen is a reservoir of monocytes that can migrate to the damaged area of the myocardium in response to acute ischemia [7]. Synchronic and timely monocyte migration ensures adequate myocardial regeneration and prevents the development of an inadequate prolonged inflammatory response with a poor prognosis in experimental and clinical conditions [8]. Continuous neurohumoral activation of the cardiosplenic axis and mobilization of pro-inflammatory splenic monocytes can occur with subsequent migration to the periphery, including myocardial tissue. Experimental animal studies [5,9] show that these events induce the progression of the inflammatory process in the myocardium to a chronic phase, which is followed by the development of adverse LVR [10]. The obtained experimental data are a good fundamental prerequisite for studying intercellular relationships in the cardiosplenic axis in MI patients. Nevertheless, a number of limitations should be considered, including a number of interspecies differences in the histoarchitecture of the spleen between humans and animal [10]. In addition, data on the human spleen structure and its pathology-related changes, including MI, are insufficient and inconsistent, since its cellular composition and shape can quickly change depending on the functional state of the body [11,12]. No studies have been performed to evaluate changes in the morphometric parameters of the spleen in patients with fatal MI, and the histoarchitecture of its functional areas during different periods of MI. More data are also needed in regard to changes in the composition of splenic macrophages depending on the period of MI, its relationship with the composition of myocardial macrophages, and the unfavorable course of MI. Deeper understanding is need about the features of spleen involvement in post-infarction myocardial remodeling, including both structural changes in the organ and the mechanisms underlying macrophage cell interaction along the cardiosplenic axis. Such knowledge will enhance the development of methods for personalized timely diagnosis and treatment of post-infarction HF.

The purpose of this study is to assess the features of spleen structure in patients with fatal MI, and to evaluate the dynamics of macrophage infiltration of the spleen and its relationship with cardiac macrophage infiltration and unfavorable outcomes.

## 2. Materials and Methods

The criterion for inclusion in this study was fatal MI type 1. We analyzed spleen fragments obtained from a study group of patients (*n* = 30) during autopsy. In addition, we analyzed myocardium fragments both from the infarction area (IA), peri-infarction area (peri-IA), and non-infarct area. These materials also were taken during autopsy and used for histological examination. The criteria for exclusion were MI types II–V, infectious complications (sepsis, pneumonia), oncological diseases, and severe valve diseases [13]. The Biomedical Ethics Committee of Cardiology Research Institute, of Tomsk National Research Medical Center (protocol No. 128) approved this study, and it was conducted in accordance with the ethical principles stated in the Declaration of Helsinki. Autopsies were performed according to the order of the Ministry of Health of the Russian Federation dated June 6, 2013, No. 354n. Signed informed consent was not obtained from the patients, which does not conflict with the ethical principles stated in the Declaration of Helsinki (“informed consent”, para. 32).

Autopsy was done during 24 h after registration of the patient’s death. We fixed the material in 10% buffered formalin during the day. The material for histological examination was prepared by the standard method using a Thermo Scientific Excelsior AS histological wiring machine (Thermo Fisher Scientific, Charleston, SC, USA), then it was embedded in paraffin using a Tissue-Tek^®^ TEC™ 6 embedding console system (Sakura, Japan). Paraffin blocks had been archived for 6 years.

### 2.1. Morphological Examination

Microscopic characteristics of the spleen were assessed using one section with a thickness of 5–6 μm cut with the HM 355S rotary microtome from each block (Thermo Fisher Scientific, Waltham, MA, USA) and stained with hematoxylin and eosin (BioVitrum, Novosibirsk, Russia) using Leica ST5010 Autostainer XL for histological staining of tissue samples (Leica, Wetzlar, Germany). Cover slipping was performed using a ClearVue machine (Thermo Fisher Scientific, Waltham, MA, USA). Histological preparations were made in accordance with the manual for the operation of automatic machines provided by equipment suppliers.

Two independent researchers performed morphological examination of the spleen via routine light microscopy using an Axio Imager M2 microscope (Carl Zeiss, Jena, Germany). An Axiocam 506 color camera (Carl Zeiss, Jena, Germany) was used to obtain microphotographs of histological preparations. In the first stage, the macroscopic characteristics of the study sample were assessed. The following parameters were evaluated: organ dimensions (length, diameter); the consistency of the organ (dense, elastic, flabby); the state of the capsule (smooth/wrinkled, thickening, color); the state of the parenchyma across the section (color, scrap: predominance of blood or pulp). The data were obtained retrospectively by analyzing the case histories and autopsy reports of deceased patients.

**Figure 1:**
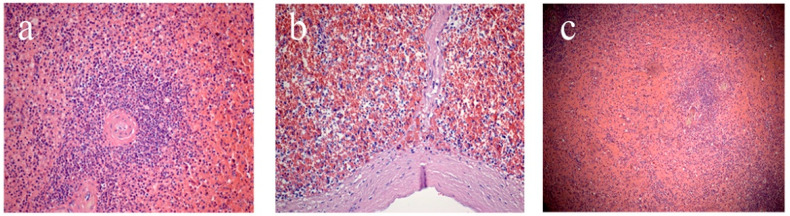
Splenic sections: (**a**) white pulp follicle, (**b**) subcapsular zone, (**c**) marginal zone. Staining with hematoxylin and eosin. Magnification ×200 (**a**,**b**), ×100 (**c**).

The second stage involved assessment of microscopic characteristics, which included analysis of the state of the capsule (thickened/not thickened, the presence of hyalinosis) and trabeculae (thickened/not thickened, the presence of hyalinosis); determination of the white pulp (WP)/red pulp (RP) ratio; as well as characterization of WP follicles (size, presence or absence of generative centers, severity of the marginal zone), and the state of the central arteries of follicles (the walls are thickened/not thickened, the presence of hyalinosis) (Figure 1). The study of the RP assessed the state of the reticular stroma (sanguineous/non-sanguineous, the presence of erythrocytes, macrophages, plasmocytes) and sinusoids (sanguineous/non-sanguineous).

### 2.2. Immunohistochemical Study

For immunohistochemical studies, microtome sections of the spleen and myocardium were prepared using an HM 355S rotary microtome (Thermo Fisher Scientific, USA). From each block, 10 sections of spleen fragments and 20 sections of the myocardium were obtained. The material was applied to glasses coated with L-polylysine (two sections per glass). Two independent researchers investigated the macrophage infiltration of the spleen and myocardium via immunohistochemical studies conducted using an automatic immunostainer (Leica Bond-Max, Wetzlar, Germany). Macrophage immunophenotyping was performed using mouse monoclonal antibodies for the common macrophage marker CD68 (Cell Marque, dilution 1:500), and antibodies for the M2 macrophage marker synthesized in the Laboratory of Innate Immunity and Immunological Tolerance (University of Heidelberg) against stabilin-1 (dilution 1:1000) [14].

**Figure 2:**
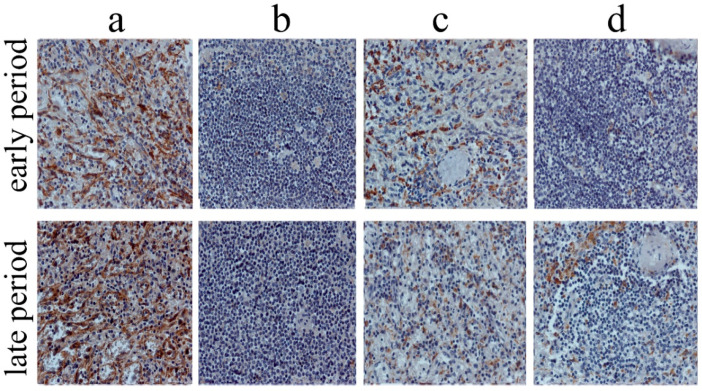
Dynamics of splenic CD68+ and stabilin-1+ cells according to the period of MI, immunohistochemistry, scale-bar 50 μm. (**a**) stabilin-1+ cells (splenic RP), (**b**) stabilin-1+ cells (splenic WP), (**c**) CD68+ cells (splenic RP), (**d**) CD68+ cells(splenic WP). Abbreviations: MI—myocardial infarction, RP—red pulp, WP—white pulp.

The studied markers were visualized using the HRP-DAB system (horseradish peroxidase-3, 3′-diaminobenzidine, peroxidase-3, 3′diaminobenzidine). Immunohistochemical staining was performed in accordance with the standard protocol [14]. Two independent researchers counted cells in both organs in 10 randomly chosen fields of view (40× objective) using a Zeiss Axio Imager M2 microscope, bright field (Figure 2).

### 2.3. Clinical and Anamnestic Data

The patients were divided into 2 groups according to the time of death. The first group included patients who died within the first 3 days after the onset of MI, while the second group included patients with fatal outcome within 4 to 28 days after MI. We have already presented clinical and anamnestic data on these groups of patients [13]. It should be noted that the age of the examined patients was 74.8 ± 9.8 years. Anterior-inferior MI was recorded in 40% of the cases, half of the patients showed recurrent MI, and 50% of the patients had history of HF. Cardiogenic shock was the most common cause of death.

We used the software STATISTICA 12.0 for the analysis of the data. A Shapiro–Wilk test was used for testing the normality of quantitative data. The quantitative indicators that did not have a normal distribution were described by using the median (Me) and interquartile range (Q1; Q3), except for age, which was described using the mean (M) and standard deviation (SD). Categorical indicators were described using frequencies and percentages. The Mann–Whitney test was used for comparison of the quantitative indicators in the groups, while χ^2^ (Pearson’s test) and Fisher’s tests were used for comparison of categorical indicators. Correlations between the concentration of cells and clinical and anamnestic data were identified using Spearman’s correlation coefficient. A value of *p* = 0.05 was used to confirm the significance of the hypotheses considered. The multivariate logistic regression model included the numbers of the studied cells CD68+ and stabilin-1+; in both the myocardium and the splenic RP and WP, as well as the presence of LV aneurysm associated with the number of these cells. The model was used to determine the cell phenotype, and its localization correlated with the formation of LV aneurysm.

## 3. Results

In the study sample, 87% of the patients had ST-segment elevation MI upon admission to the hospital, and in 65% of the cases, it had occurred for the first time. More than one third of the examined patients had a lethal outcome during the first day after the onset of the disease. Table 1 shows the main macroscopic characteristics of the spleen in patients with fatal MI. It should be noted that the norms used to assess these parameters [15] were conventional, since the mass, volume, and size of the spleen can vary significantly depending on the venous blood storage and the activity of hemostasis.

The spleen length in patients with fatal MI and its average thickness were slightly less than the norm (N). However, the analysis showed that the spleen length in 29% of cases (*n* = 9) was greater than N, while in 14% (*n* = 4), it corresponded to N, and in 57% (*n* = 17), it was less than N. The spleen width was less than N in 42% (*n* = 12) of the cases, and in other patients, the spleen thickness slightly exceeded N. The thickness was within the normal range in 86% (*n* = 26), and was reduced in 14% (*n* = 4). The mass in 71% (*n* = 21) of the cases was less than N, and in 29%, it exceeded N. Interestingly, in patients with reduced spleen mass, the spleen length was also reduced.

When assessing the microscopic characteristics of the spleen, WP and RP indicators were analyzed (Table 2).

The results thus far indicate that the splenic RP predominates regardless of the period of MI. In addition, medium and large follicles predominated in the WP. Immunohistochemical study of splenic and myocardial tissues in patients with fatal MI revealed both stabilin-1+ and CD68+ cells in tissues of both organs. Their dynamics are presented in Table 3.

**Figure 3:**
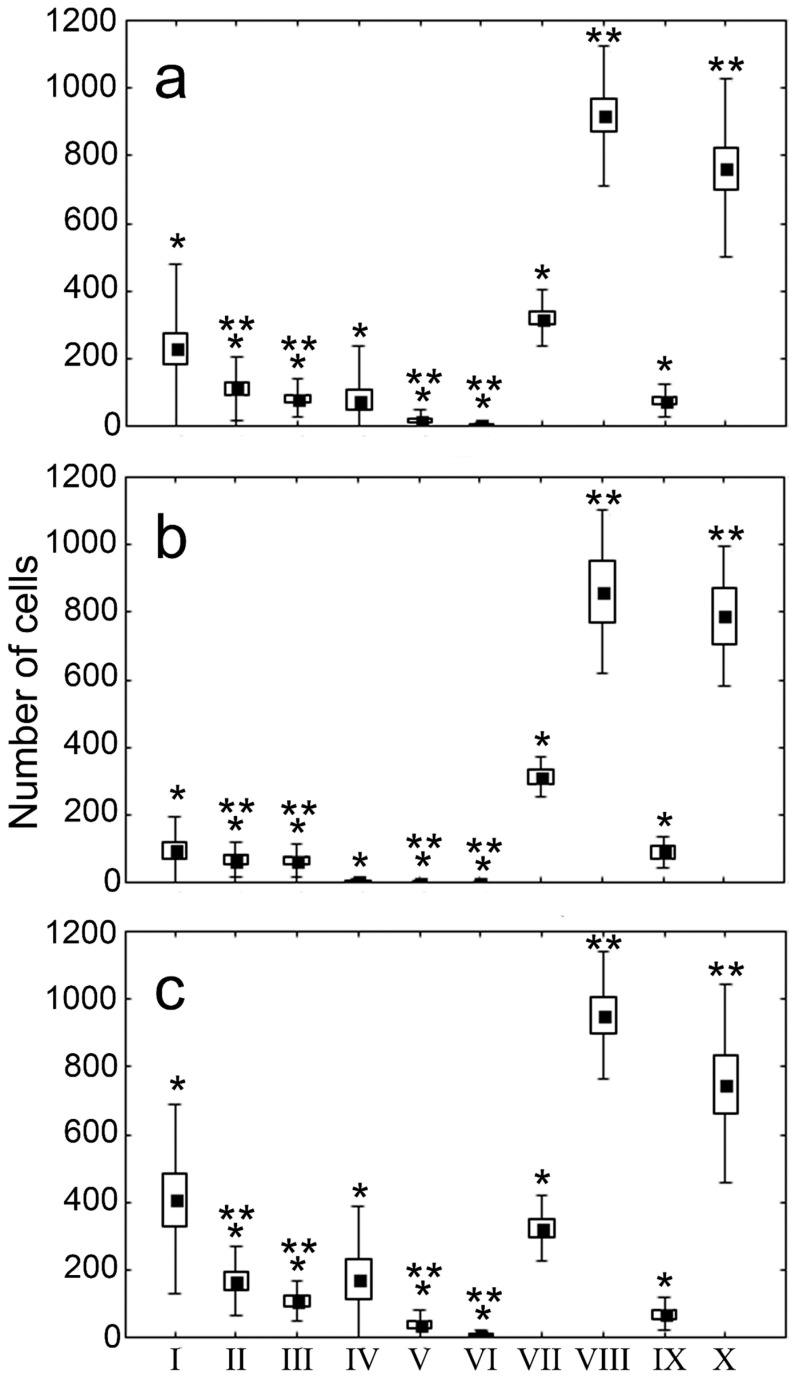
The percentage of CD68+ and stabilin-1+ cells depending on the period of MI. I—CD68+ cells (IA), II—CD68+ (peri-IA), III—CD68+ (non-IA), IV—stabilin-1+ cells (IA), V—stabilin-1+ (peri-IA), VI—stabilin-1+ (non-IA), VII—CD68+ (WP), VIII—CD68+ (RP), IX—stabilin-1+ (WP), X—stabilin-1+ (RP). Abbreviations: IA—infarct area, RP—red pulp of the spleen, WP—white pulp of the spleen. (**a**) the diversity of the cells in all patients, (**b**) in group 1, (**c**) group 2. Note: *—statistically significant difference from the cells in red pulp of the spleen, **—statistically significant difference from the cells in white pulp of the spleen and infarct area of myocardium.

Intensive infiltration in both types of cells was characteristic of the RP of the spleen. These cells were found to significantly dominate in comparison with myocardial macrophages and macrophages in the WP. The number of CD68+ and stabilin-1+ cells in the WP of the spleen and in the IA of the myocardium was comparable, but their number in the peri-IA and non-IA was significantly lower (Figure 3).

Interestingly, the concentration of CD68+ and stabilin-1+ cells in the myocardium increased by the late period of MI, while no such dynamics were found for the spleen.

**Figure 4:**
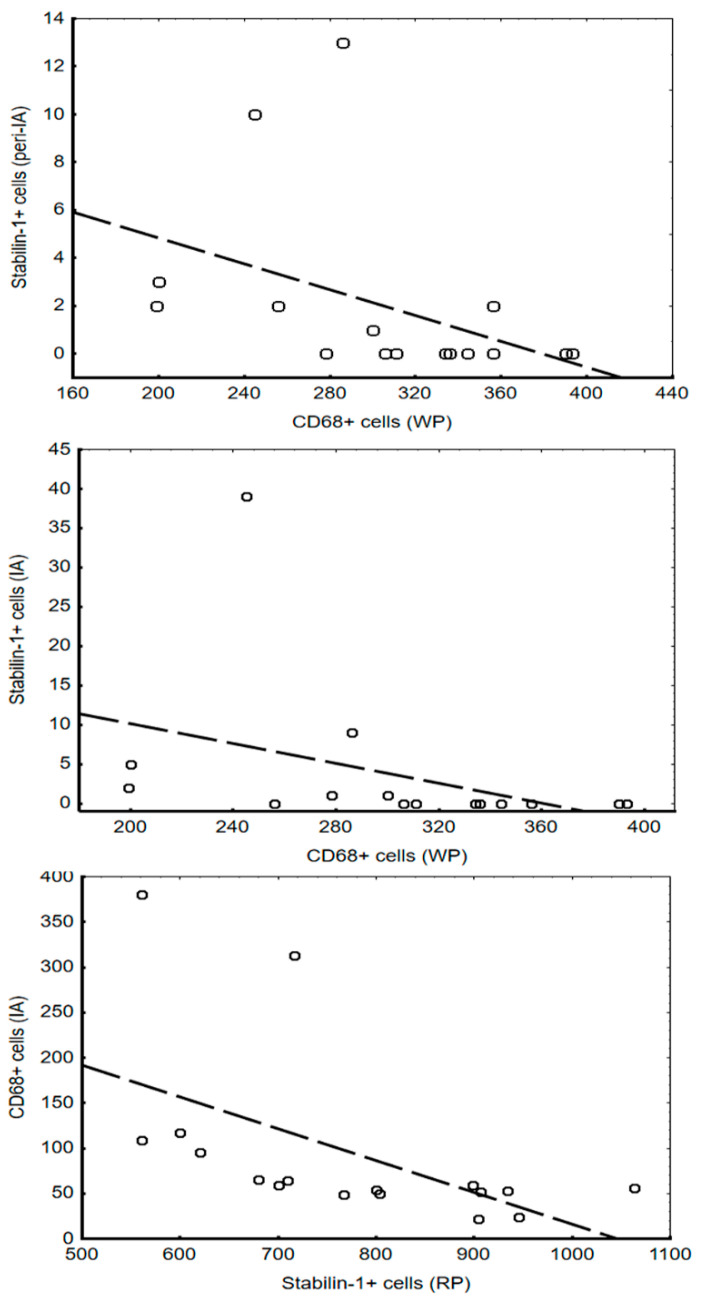
Correlations between CD68+ and stabilin-1+ cells in the spleen and myocardium. Abbreviation: IA—infarct area, RP—red pulp, WP—white pulp.

Analysis of the associations between the number of investigated cells in the myocardium and spleen showed that these correlations are found only in the early period of MI (Figure 4).

In addition, the percentage of stabilin-1+ cells in the WP of the spleen correlated with the presence of LV aneurysm and preinfarction angina (r = −0.6; *p* = 0.03). However, the percentage of CD68+ cells in the RP of the spleen (r = 0.7; *p* = 0.04) showed a direct correlation with these indicators. All the cells analyzed in both the spleen and the myocardium, were used for the multivariate analysis model to assess the cell type associated with the formation of LV aneurysm (Table 4). It was found that the concentration of CD68+ cells in the RP was related to the formation of LV aneurysm (β = 0.9, *p* = 0.04).

## 4. Discussion

Currently acute myocardial ischemia and cardiomyocytes’ death that occur during MI are considered to trigger a two-phase inflammatory response in the damaged area of the myocardium [16,17]. Cells of the immune system (monocytes/macrophages) are key participants in this process [18,19]. Circulating monocytes migrate to the affected area (myocardium) with the blood flow and differentiate into M1 macrophages of the pro-inflammatory phenotype [20]. Starting from day 3, under the impact of the microenvironment, M1 polarizes to M2 macrophages, and the processes of myocardial regeneration are triggered [16]. The regeneration phase is followed by the formation of scar tissue [6,18], and then, 2 weeks after MI, the percentages of monocytes and macrophages in the damaged area of the myocardium return to the initial level. In the non-IA of the myocardium, macrophages may persist for months [21]. Timely resolution of inflammation and polarization of M1 to M2 macrophages are prerequisites for a favorable course of post-infarction myocardial regeneration. Timely and synchronous migration of leukocytes and monocytes to cardiac tissue, along with the coordinated activity of these cells in myocardial tissue, provides adequate myocardial regeneration and prevents an inadequate protracted inflammatory response, which has shown an unfavorable prognosis in experimental and clinical conditions [16,22].

The spleen is one of the major reservoirs of leukocytes, including monocytes, which migrate to myocardial tissue in response to acute ischemic injury [23]. It is a large lymphoid organ, that performs filtration, cleansing, and hematopoietic functions, as well as storage and immune functions [24]. In 2009, Swirski et al. were the first to focus on the relevance of cellular-molecular processes occurring in the cardiosplenic axis under ischemic conditions [7]. They revealed that activation of the bone marrow in rodents under myocardial ischemic conditions and activation of the sympathetic nervous system increase the number of hematopoietic stem cells and bone marrow progenitor cells. These cells migrate to the spleen and populate it, and the spleen becomes a focus of active extramedullary hematopoiesis. Then, monocytes mobilize from the spleen under the impact of increased content of angiotensin II in plasma and actively infiltrate the damaged ischemic myocardium within 1 day. Experimental data indicate that this global immune activation of the spleen and bone marrow under ischemic conditions ultimately leads to splenomegaly and remodeling of splenic tissue [10]. The pool of CD4+, CD8+ macrophages of a pro-inflammatory phenotype, dendritic cells, and T cells (Th1, Th2, Th17, and Treg) in the WP increase, while the pool of mononuclear cells in the RP decreases. Activated immune spleen cells entering myocardial tissue cause local damage, cell death, and cardiac fibrosis, which contribute to the development of adverse heart remodeling [5,9,25].

Interestingly, in the study sample, the concentration of these cells in the splenic WP and RP did not change, but their number in the myocardium increased and was associated with an adverse outcome. This partly confirms the results of experimental studies, but to determine whether the number of splenic macrophages increases in response to MI, it is necessary to compare their numbers in patients without cardiovascular pathology in future research. The experiment on mice showed that splenectomy [9] or CCR2 knockout decreased myocardial infiltration by Ly-6Chigh cells, (analogues of pro-inflammatory macrophages in humans), which was followed by an increased LV ejection fraction and a decreased LV end-diastolic volume.

In 2015, Emami et al. studied the involvement of the spleen in post-infarction myocardial remodeling in humans. They observed increased splenic metabolic activity after acute coronary syndrome (ACS) and its relationship with pro-inflammatory remodeling of circulating leukocytes. They also observed high prognostic value of increased spleen activity after MI and the development of subsequent cardiovascular events [23]. However, these data were obtained in vivo from patients with ACS and coronary artery disease, and then they were compared with those in healthy patients from the control group using scintigraphy. Another study based on autopsy material [26] assessed the content of CD14+ monocytes and their subtypes in the myocardium, spleen, and bone marrow in patients with ACS. The study showed that the recruitment of monocytes to the myocardium is accompanied by the depletion of their pool in the spleen. Our results obtained in regard to the correlations between stabilin-1+ cells in the WP and in the myocardium confirm these data. In addition, we assessed the dynamic changes in the pool of both pro- and anti-inflammatory types of macrophages according to the period of MI and their relationship with clinical and anamnestic data, as well as with each other. These results contribute to the knowledge about the composition of splenic macrophage infiltration in patients with MI, its dynamics according to the period of MI, and the relationship with macrophage infiltration of the myocardium and adverse outcomes.

Furthermore, we assessed the size and the micro- and macroscopic characteristics of the spleen in patients with fatal MI. It was found that indicators such as the length and mass of the spleen were reduced in most patients. These data are contradictory since similar data previously obtained in an experiment on rodents were attributed to the depleted pool of macrophages during MI [16]. Yet there are data on the development of splenomegaly in rodents after MI [10] due to global immune activation of the bone marrow and spleen. However, these data can reflect the anthropometric characteristics of the patients, and changes in splenic tissue can be associated with well-studied pathological mechanisms that are directly related to the progressive chronic HF (CHF) in patients with recurrent MI [27].

Chronic venous congestion is known to develop in patients with CHF, in both the pulmonary and systemic circulation; thus accompanied by characteristic morphological changes in the organs, including primarily atrophy and replacement of the parenchyma by connective tissue. This remodeling ultimately decreases the parenchyma volume, and reduces the size and mass of the organs. To understand how these changes reflect the activation of the cardiosplenic axis under ischemic conditions, they should be analyzed in patients who survived after MI.

The analysis of the microscopic characteristics revealed a predominance of the RP performing mainly a filtration function, which may indicate excessive venous blood storage in the spleen [12]. The quantitative content of the WP involved in the functioning of the immune system was lower, and numerous follicles of medium and small sizes predominated, which were comparable with previously obtained data [7]. Our results also contribute to the pool of data on post-infarction changes in splenic tissue in patients with MI, and could motivate further studies, since no systematic data are available on changes in the morphological characteristics of the spleen in response to different cardiological pathologies [11].

We assessed the percentage of CD68+ and stabilin-1+ cells when analyzing macrophage infiltration of the spleen and myocardium. The CD68+ marker is most frequently used in oncohematology and is involved in the detection of the general population of macrophages, but it has also been reported to have a significant role in cardiology [18,28]. Stabilin-1+ is a marker of M2 macrophages and expressed on tissue macrophages and sinusoidal endotheliocytes of the spleen, lymph nodes, and liver [14]. According to our previous results, stabilin-1+ cells are involved in post-infarction myocardial remodeling, but its content was assessed only in myocardial tissue.

In 1987, Buckley revealed different types of macrophages and dendritic cells in the spleen [29]. However, there has been no evaluation of the pool of pro- and anti-inflammatory macrophages and their dynamics in the myocardium and the spleen according to the period of MI. We found cells with the pro-inflammatory phenotype of CD68+ and anti-inflammatory cells stabilin-1+ cells in both organs. However, the highest percentage of these cells was found in the splenic RP, which may be due to its main functions of filtration and purification of the blood [15]. It is known that 15–20% of the blood volume circulates through the spleen at any given time, and about 15% of lymphocytes are permanently located in this organ [30]. Due to an extensive arterial network in the RP, the number of macrophages can rapidly increase as a result of the migration of monocytes from the blood and their further differentiation [31].

During experimental MI, about 40% of splenic monocytes involved in healing of the damaged area of the myocardium were found in the focus of inflammation [7]. The number of macrophages observed in the splenic WP was smaller than in the RP, but comparable to the number of macrophages in the IA of the myocardium. This confirms the experimental data and indicates that the spleen, particularly the WP, is a reservoir of monocytes migrating to the damaged area of the myocardium during MI [7]. In the peri-IA and non-IA of myocardium, the number of these cells was significantly lower, which confirms this hypothesis.

Interestingly, the increase of the macrophage concentration in the infarcted myocardium, and in the later period of MI in patients with a fatal outcome, did not reduce the number of these cells in the spleen. Our data partly correspond to previously published results [26] and indicate that after MI, splenic monocytes that contribute to the repair of the affected area accumulate in the myocardium. However, experimental data show that their migration coincides with a decrease of the concentration of these cells in the spleen [23], which was not observed in our sample. The number of splenic macrophages is not likely to decrease despite the healing processes that start in the IA of the myocardium. Furthermore, active inflammation is followed by incipient regeneration on day 3 after injury, which could have caused an adverse outcome in patients. The remaining high content of stabilin-1+ cells indicates a favorable course of regeneration, whereas the remaining high content of CD68+ cells indicates active inflammation, which does not contribute to myocardial regeneration.

Analysis of the relationship between the macrophage pools in the spleen and myocardium showed that these indicators correlate only in the early period of MI. This is probably due to the highest monocyte migration from the spleen to the myocardium occurring in only the first 24 h. A high percentage of CD68+ cells in the IA of myocardium associated with a lower percentage of stabilin-1+ cells in the splenic RP and characterizes the early period of MI, which involves active inflammation. A high concentration of CD68+ cells in the splenic RP was due to the formation of LV aneurysm, as was the low concentration of stabilin-1+ cells in the WP to a lesser extent. This can be promoted by persistent active inflammation and decreased regenerative activity in the myocardium.

Undoubtedly, the study had a number of limitations. The study sample was limited in volume, and the ratio of splenic/myocardial macrophages at a given time period was not a static indicator. Therefore, a promising direction for future research is the changes in the amount of cells with these phenotypes in vivo. This is supported by the ability of the spleen to change structure quickly depending on the functional state of the body, and autolytic processes begin immediately after death [11].

Our data provide insight on changes in macrophage infiltration of the spleen under ischemic conditions and their relationship with the alterations of the macrophage infiltration of the heart, as well as the development of an adverse outcome in MI patients. Such information could shed light on the mechanisms of formation and progression of the inflammatory response in the myocardium. This could enhance the development of treatment methods for local control of the intensity and duration of the response. There has been no success in attempts to influence the inflammatory response after MI through available therapeutic strategies to suppress inflammation. The current therapeutic modes have shown no beneficial effect, and there is no prognostic evidence of improved outcome of the disease in this patient cohort. Such difficulties are most likely due to the insufficient knowledge about the mechanisms of cell-stromal interactions, as well as the molecular genetic features of the regenerative processes in patients with MI. These could become a potential target for personalized and controlled therapy aimed at activating and controlling the intensity of pro- and anti-inflammatory responses during development of post-infarction HF, as well as regulating their balance.

## 5. Conclusions

The spleen of the patients with fatal MI was decreased and showed a predominance of red pulp with a high concentration of CD68+ and stabilin-1+ cells. The white pulp contained many medium and small follicles, and a lower concentration of CD68+ and stabilin-1+ cells, which was comparable to that in the infarct area of the myocardium. The concentration of CD68+ and stabilin-1+ cells increased in the myocardium in the late period of MI, but did not show any dynamics in the spleen. A high number of CD68+ cells in the red pulp and reduced concentration of stabilin-1+ cells in the white pulp were associated with unfavorable post-infarction outcomes. These fundamental findings could be a basis for the development of new personalized therapeutic and diagnostic approaches for the treatment of MI and its complications.

## Figures and Tables

**Table 1:** Macroscopic characteristics of the spleen in patients with fatal MI (*n* = 30).

Parameters	N [15]	General Group (*n* = 30)	Group 1 (*n* = 17)	Group 2 (*n* = 13)	*p*
Length, mm	110–130	110 (100; 180)	125 (110; 180)	105 (100; 132)	0.2
Width, mm	75	80 (10; 85)	60 (10; 85)	80 (70; 80)	0.7
Thickness, mm	25	30 (30; 45)	30 (20; 60)	30 (30; 37)	1.0
Consistency of the spleen: elastic, % (*n*)/ flabby, % (*n*)	-	40% (12)/ 60% (18)	30% (5)/ 70% (12)	40% (5)/ 60% (8)	1.0
Mass of the spleen, gm	150–200	130 (113; 427)	155 (128; 427)	113 (113, 133)	0.2
State of the capsule: smooth, % (*n*)/ wrinkled, % (*n*)	-	80% (24)/ 20% (6)	100% (17)/ 0	70% (9)/ 30% (4)	0.5

Abbreviations: N—normal range.

**Table 2:** Microscopic characteristics of the spleen in patients with fatal MI (*n* = 30).

Parameters	General Group (*n* = 30)	Group 1 (*n* = 17)	Group 2 (*n* = 13)	*p*
Capsule: thickened/ not thickened	2 (7%)/ 28 (93%)	2 (12%)/ 15 (88%)	0/ 13 (100%)	0.3
Trabeculae: thickened/ not thickened	3 (10%)/ 27 (90%)	2 (12%)/ 15 (88%)	1 (8%)/ 12 (92%)	1.0
Pulp ratio (RP:WP): 1:1/2:1/3:1	3 (10%)/ 14 (47%)/ 13 (43%)	2 (12%)/ 5 (29%)/ 10 (59%)	1 (8%)/ 9 (69%)/ 3 (23%)	0.8
Follicles: numerous/ few	5 (17%)/ 25 (83%)	5 (29%)/ 12 (71%)	0/13 (100%)	0.3
Follicle size: small/medium/ large	9 (30%)/ 14 (47%)/ 7 (23%)	6 (35%)/ 6 (35%)/ 5 (30%)	3 (23%)/ 8 (62%)/ 2 (15%)	0.8
Follicle size per 5 pieces, mm	0.26 (0.2; 0.48)	0.25 (0.2; 0.46)	0.28 (0.24; 0.48)	0.1
Light centers: yes/no	0/30 (100%)	0/17 (100%)	0/13 (100%)	1.0
Mantle zone: pronounced/ not expressed	5 (17%)/ 25 (83%)	2 (12%)/ 15 (88%)	3 (23%)/ 10 (77%)	0.7
Follicle central arteries: thickened/not thickened	15 (50%)/ 15 (50%)	10 (62%)/ 7 (38%)	5 (45%)/ 8 (55%)	0.5
RP: desolate/ plethoric/ moderately plethoric	5 (17%)/ 20 (66%)/ 5 (17%)	4 (24%)/ 9 (52%)/ 4 (24%)	1 (8%)/ 11 (84%)/ 1 (8%)	1.0
Fibrosis of the reticular stroma: yes/no	21 (70%)/ 9 (30%)	12 (71%)/ 5 (29%)	9 (69%)/ 4 (31%)	0.9
Hemosiderosis: yes/ no	22 (73%)/ 8 (27%)	13 (76%)/ 4 (24%)	9 (69%)/ 4 (31%)	0.8
Sinusoids: sanguineous/ non-sanguineous	4 (13%)/ 26 (87%)	3 (18%)/ 14 (82%)	1 (8%)/ 12 (92%)	0.6

Abbreviations: RP—red pulp, WP—white pulp.

**Table 3:** Dynamics of the splenic and myocardial macrophages according to the period of MI (*n* = 30).

Parameters	General Group (*n* = 30)	Group 1 (*n* = 17)	Group 2 (*n* = 13)
Splenic CD68+ (RP)	898 (365; 1318)	884 (365; 1076)	912 (670; 1318)
Splenic CD68+ (WP)	312 (199; 550)	334 (199; 393)	312 (204; 550)
Cardiac CD68+ (IA)	106 (56; 376)	59 (52; 95)	376 (136; 634) *
Cardiac CD68+ (peri-IA)	78 (44; 154)	48 (36; 83)	154 (85; 232) *
Cardiac CD68+ (non-IA)	67 (38; 115)	44 (33; 75)	95 (61; 141) *
Splenic stabilin-1+ (RP)	746 (410; 1201)	807 (561; 1063)	708 (410; 1201)
Splenic stabilin-1+ (WP)	59 (7; 174)	86 (30; 140)	56 (7; 174)
Cardiac stabilin-1+ (IA)	1.5 (0; 102)	0 (0; 1)	126 (42; 216) *
Cardiac stabilin-1+ (peri-IA)	1 (0; 13)	0 (0; 2)	24 (1; 70)
Cardiac stabilin-1+ (non-IA)	0 (0; 3)	0 (0; 0)	0 (0; 13)

Abbreviations: IA—infarct area, RP—red pulp, WP—white pulp. Note: *—significant difference between the groups.

**Table 4:** Multivariate linear analysis. Correlations between the formation of LV aneurysm and the number of splenic and myocardial cells (CD68+, stabilin-1+) in patients with fatal outcome (*n* = 30).

Parameters (Cells)	Β (Standardized Deviation)	*t*-Value	*p*
Cardiac CD68+ (IA)	−0.4	−0.9	0.3
Splenic CD68+ (RP)	0.9	2.4	0.04
Splenic CD68+ (WP)	−0.5	−1.5	0.2
Cardiac stabilin-1+ (IA)	0.4	0.7	0.5
Splenic stabilin-1+ (RP)	0.2	0.7	0.5
Splenic stabilin-1+ (WP)	0.2	0.7	0.5

Abbreviations: IA—infarct area, LV—left ventricular, RP—red pulp, WP—white pulp.
